# Kidney replacement therapies in the older person: challenges to decide the best option

**DOI:** 10.1093/ckj/sfaf020

**Published:** 2025-01-22

**Authors:** Jessica Selwood, Melanie Dani, Richard Corbett, Edwina A Brown

**Affiliations:** Department of Renal Medicine, Hammersmith Hospital, London, UK; Department of Geriatric Medicine, Hammersmith Hospital, London, UK; Department of Renal Medicine, Hammersmith Hospital, London, UK; Department of Renal Medicine, Hammersmith Hospital, London, UK

**Keywords:** age, haemodialysis, peritoneal dialysis, shared decision making, supportive care

## Abstract

A multitude of challenges exist when supporting older adults in deciding on the optimal kidney replacement therapy (KRT), including frailty, comorbidity, cognitive impairment, dialysis modality, as well as local availability of services. The combination of these factors can determine treatment outcomes and quality of life (QoL), and as such the care of older people should be tailored to take these into account. Frailty in older people with chronic kidney disease (CKD) leads to higher rates of hospitalization, increased mortality, and a diminished QoL, while cognitive impairment, present in up to 50% of people with CKD, exacerbates these challenges and affects decision making.

Dialysis, particularly haemodialysis, can accelerate physical and cognitive decline in frail older adults. Conversely, peritoneal dialysis (PD) presents a home-based alternative that may better support QoL, particularly for people wanting to prioritize treatment flexibility and independence. Assisted PD programmes have emerged as a valuable option for older people who cannot manage home-based care independently, improving access to KRT.

Ultimately shared decision making should be employed when discussing KRT, incorporating patient goals, prognostic awareness, and QoL measures. There is also the emerging role of the geriatrician and the need for an integrated Comprehensive Geriatric Assessment. These elements support older adults to make informed choices that align with the individuals’ values and health needs.

In designing future health services to meet the needs of increasing numbers of older people, there needs to be increased access to assisted PD as well as multidisciplinary working to ensure patient-focused care surrounding KRT in older adults.

## INTRODUCTION

On 1 September 2024, the *New York Times* published an article entitled ‘The New Old Age: Dialysis May Prolong Life for Older Patients. But Not by Much’ [1], reflecting the wider awareness in society of the challenges of delivering kidney replacement therapy (KRT) with an ageing population. Older people developing kidney failure are likely to be multi-morbid [2], and compared to the general population have a higher rate of age-related syndromes including frailty [3], falls [4], and cognitive impairment [5]. Quality of life (QoL) for older people on dialysis is mostly related to the degree of frailty [6], which is not surprising considering that poor functional status is related to worse patient related outcomes for all those on haemodialysis (HD) and peritoneal dialysis (PD) [7]. People with kidney failure who are frail have reduced likelihood of transplantation, higher short-term complications (delayed graft function, increased hospitalization), and poorer survival post-transplantation [8, 9]. Regardless of the modality chosen, survival for all types of kidney replacement therapy (KRT) is poor in older people, with a median survival in the UK of ∼3 years for all those >65 years old when starting dialysis [10]. This emphasizes the need for the fourth option—supportive care—to be incorporated through all stages of advanced kidney disease management and decision making for older people. Incorporating supportive care requires decision making around dialysis or no dialysis, and considering how each dialysis modality should be adapted to maintain well-being [11]. This review will focus on age-related challenges that should be considered when considering dialysis or transplantation, the outcomes of HD and PD and how the availability of assisted PD may affect dialysis modality choice. All these factors are essential to inform shared decision making to enable the best KRT option for the older individual.

## AGE-RELATED CHALLENGES

### Frailty and cognitive impairment: assessment and implications

Frailty—an increased vulnerability to stressors resulting from diminished physiological reserves [12]—has been reported in up to 82% of older patients with chronic kidney disease (CKD) stage 5 [13]. Identifying it correctly and considering its implications in treatment goals is fundamental. For many patients, physical and cognitive frailty dominate, and may be more burdensome than the renal disease itself. Counselling these patients about what to expect from life with frailty, cognitive impairment, and the potential impact of dialysis, is of paramount importance.

### The impact of frailty on prognosis and quality of life

The ‘frailty syndromes’ encompass cognitive impairment, poor physical mobility, incontinence, dependency for activities of daily life, and falls. These syndromes, and their significance, can be easily overlooked if they are not systematically explored [14]. The presence of frailty is associated with increased mortality across all stages of CKD [15–17]. Individuals who are frail when starting dialysis have a shorter time to their first hospitalization and increased mortality [16]. In frail individuals with CKD, gait speed (a key component of the frail phenotype) can be a more robust predictor of mortality than kidney function [18]. At an individual level, frailty in patients with CKD confers higher symptom burden and worse QoL compared to those without frailty [19]. For patients who are on HD, frailty is associated with increased hospitalization, more emergency presentations, and more vascular access events [20]. Additionally, there are higher rates of depression, falls, and fractures [20].

### The impact of cognitive impairment on prognosis and quality of life

Cognitive impairment is also common in CKD and estimated to be present in ∼40%–50% individuals and is commoner as kidney disease progresses [21, 22]. Like physical frailty, it is also associated with increased mortality [23]. The specific patterns of cognitive impairment and the affected domains differ from those in other neurodegenerative conditions, with orientation, attention, concept formation, and reasoning being most commonly affected [24]. Unsurprisingly, cognitive impairment and frailty do not exist in isolation—one study of patients with CKD stages 3–5 who were not on dialysis showed that the presence of cognitive impairment was associated with impaired mobility, muscle strength, and increased falls [25].

### The impact of dialysis on frailty and cognitive impairment

Dialysis can be a major undertaking for frail, older individuals. On a day-to-day basis, both frailty and older age are associated with longer recovery time following haemodialysis [26]. There are also changes over longer time periods: ‘physical performance’ (based on self-reported physical activity limitations) deteriorates in people on HD: one study found that in patients >75 years with poor physical function receiving HD, 58% had died at 2-year follow-up. Even in the older patients with good physical function at baseline, 42% had deteriorated functionally after 2 years, and 32% had died [27]. Patients on PD also experience acceleration of physical frailty over time, which is accelerated by co-existent nutrition, dementia, and hospitalizations [28].

### Brief overview of assessment tools

Several tools for frailty and cognitive evaluation are available. The most commonly used is the Fried Frailty Phenotype (briefly, three or more of the following criteria: unintentional weight loss, weakness measured by grip strength, exhaustion, slow gait speed, and low physical activity) [29]. This rapidly administered and intuitive tool is predictive of poor outcomes in CKD patients, especially those with more advanced disease, and can be used to identify patients who will benefit from more targeted assessment and treatment with exercise and nutritional support [30]. The Rockwood Clinical Frailty Score (CFS) is a widely used visual scale ranging from 1 to 9 based on functional ability and dependence [31]. Similarly, it has been shown to correlate with mortality within 6 months of starting dialysis [32]. The Fried phenotype remains the most commonly used tool in research studies [33]. However, this may change over time to reflect clinical practice where the CFS may be used more routinely [34].

The gold standard tool for comprehensively assessing and treating older patients is the Comprehensive Geriatric Assessment (CGA), encompassing a multidisciplinary, multidimensional assessment of the individual's medical, functional, and psychosocial state with individualized goals and recommendations for care [35]. Its use in acute hospital inpatients increases the likelihood of patients being alive and in their own home after 3 to 12 months [36]. CGA is increasingly used in patients with CKD [37, 38] and has been customized for renal patients: the CKD-CGA has been shown to predict mortality, hospitalization, and dialysis initiation in older patients [39, 40].

Cognitive impairment screening tools include the Mini Mental State Examination (MMSE), Montréal Cognitive Assessment (MOCA), Addenbrookes Cognitive Examination 3 (ACEIII), and Roland Universal Dementia Assessment Scale (RUDAS). The MMSE is historically the most utilized but has fallen out of clinical practice recently due to its limited generalisability across cultures and educational settings [41]. The MOCA is well-validated in diagnosing both dementia and mild cognitive impairment [42], and has a high predictive ability for identifying cognitive impairment in patients on dialysis [43]. Additionally, the MOCA assesses multiple cognitive domains giving a global assessment of an individual's cognition. RUDAS is less weighted by language, educational level, and cultural background, and can be useful in diverse psychosocial and cultural settings [44, 45]. There is no specific ‘gold standard’ recommended screening test, but a validated tool assessing multiple cognitive domains is required.

### The role of the geriatrician

Integrated multidisciplinary working with geriatricians is important for optimizing the non-renal aspects of an older person's presentation, as well as for considering the impact of KRT interventions in people with frailty. An intrinsic component of the CGA in the general population is multidisciplinary involvement, including a geriatrician.

The geriatrician brings a specific added skillset to this process; in one study, only 55% of the problems identified by the geriatrician were recorded in the nephrologists’ notes [46]. Similarly, nephrologists completing the CFS without receiving training had low correlation with the gold standard frailty index, suggesting a specific need for expertise over general ‘intuition’ or bedside impression [47]. A randomized control trial comparing the CGA in patients with CKD, compared with usual care, is currently underway and the focus of the GOAL trial [48].

## HD AND PD OUTCOMES

People undergoing HD and PD often have varying experiences of their treatment due to individual characteristics, comorbidity, and lifestyle [49]. Numerous studies have shown that there may not be a survival benefit to any dialysis modality in older people [50, 51], particularly in those with significant comorbidity [52]. However, recent research has explored additional QoL measures including hospitalization, maintenance of independence and treatment satisfaction. These factors are likely to be more important to the older person and as such influence their choice of KRT [53]. Table 1 summarizes the key findings of studies comparing outcomes in older people on HD versus PD, including international research [54–56] demonstrating the heterogeneity of this discussion globally. However, mortality data does not consider socio-demographic factors, for example the UK has an ageing population and a larger proportion of older people living with CKD [10]. This highlights the need for thorough consideration of QoL measures via shared decision making when reviewing older patients.

**Table 1:** Some key studies comparing outcomes of older patients on HD and PD.

		Study population (*n*)		
Study	Age of study participants (years)	HD	PD	Key findings
North Thames Dialysis Study Harris *et al*. [50]	>70	96	78	• No difference in mortality or QoL at 12 months
BOLDE study Brown *et al*. [57]	>65	70	70	• QoL not statistically different between HD and PD cohorts • Depression and feelings of illness intrusion significantly less in the PD cohort
ERA–EDTA Registry van de Luijtgaarden *et al*. [54]	Multiple age categories, including 60–69 and >70	8347	1544	• Survival better on PD in patients >70 years versus HD • Increasing age associated with a lower likelihood of receiving PD, particularly in females • Non-diabetic patients and those with malignancy less likely to receive PD, despite better survival rates
Foote *et al.* [52]	>75	1328	453	• Median survival for the older person starting dialysis was 2.3 years • Mortality risk increased with number of comorbidities and age • Late referral and lack of prepared dialysis access were risk factors for poorer survival
Korean meta-analysis Han *et al*. [55]	>65	10 675	2390	• Higher mortality rate in PD cohort compared to HD (*P *< .001) • Survival benefit of HD in those with diabetes mellitus or dialysis duration >1 year
Wolfgram *et al.* [68]	mean = 69.2	112 960	8663	• Lower incidence of dementia in the PD cohort over 3 years • Risk of dementia lower for people starting on PD
FEPOD study Iyasere *et al*. [6]	>60	122	129 (assisted PD)	• No difference in QoL • Higher treatment satisfaction scores for people on assisted PD compared to those on in-centre HD
Swedish Renal Registry Rydell *et al*. [56]	>65	118	118 (assisted PD)	• No significant difference in number of days per year in hospital, number of hospitalizations, or discontinuation of dialysis modality between groups • Worse survival on assisted PD, however likely due to use as a palliative treatment • Assisted PD likely to be a reasonable alternative to HD in the frail, older population

One of the first studies to examine dialysis outcomes in an older cohort was the BOLDE study (Broadening Options for Long-term Dialysis in the Elderly) [57], which focused on patients >65 years, including 70 individuals treated with both PD and HD respectively. QoL was not statistically different between the two groups, however, the study found that depression and feelings of illness intrusion were significantly less in the PD cohort compared to those on HD. This may be of relevance given that depression is not only prevalent in the older population, but increasingly difficult to recognize due to the clinical predominance of somatic symptoms [58]. Thus, maintenance on a home-based treatment that patients find less invasive may be beneficial for older people and contribute to overall improved mental well-being, which is a key component of healthy ageing [59].

Frailty, as discussed previously, is integral when discussing options for KRT, and its impact was considered in the FEPOD (Frail and Elderly Person on Dialysis) Study [6]. Matched cohorts of people aged >60 years with over 120 individuals treated with HD and assisted PD respectively were examined using assessments of QoL. This study showed that treatment satisfaction was higher in the assisted PD group compared to those on HD, but otherwise there was no differences related to dialysis modality. Indeed, frailty was the predominant predictor of all the patient reported outcome measures with worse scores associated with increasing frailty. As well as adversely affecting an older person's experience of dialysis, frailty is associated with an increase in adverse events, such as falls, fractures, hospitalization, cardiovascular events, and death for those on PD and HD [60]. Declining physical function and increasing frailty in older frail individuals after starting dialysis has been recognized for some years [61, 62] and is associated with higher mortality risk [63].

Dialysis also affects progression of cognitive impairment. In patients on HD, there is an annual deterioration in MMSE score [64]. There also appears to be a disproportionate impairment in executive function over time [65]. While there are limited studies showing a decline in cognition on PD, the prevalence of cognitive impairment is high in these patients [66], and even patients receiving PD who have intact cognition have altered brain structure compared to controls [67]. A retrospective cohort study by Wolfgram *et al.* [68] of >120 000 patients incident to HD and PD demonstrated a lower incidence of dementia in the PD cohort over a mean follow-up time of 1.5 years. The mechanism behind this may relate to changes in cerebral physiology that occur during haemodialysis sessions. Reduced cerebral oxygen saturation during HD [69] was associated with global worsening cognition in a cohort of 24 patients. These findings were further supported by a prospective observational cohort study of 97 haemodialysis patients [70] using various imaging modalities alongside testing of cognition to determine the longer-term effects of HD. A reduction in cerebral blood flow was seen during HD and for up to 30 minutes following the cessation of dialysis, alongside impaired executive function during HD sessions. Over the longer term (12 months), MRI scans revealed structural changes including cerebral atrophy as well as white matter changes signifying small vessel disease.

These findings underline the importance of CGA, including but not limited to an individual's baseline cognitive state. Informed shared decision making should include discussions around how any pre-existing cognitive impairment may deteriorate over time with haemodialysis. In comparison, PD avoids this burden of haemodynamic stress, and thus may be a more suitable treatment for the older person considering dialysis, particularly in those with pre-existing dementia. Conversely, underlying cognitive impairment may necessitate in-centre haemodialysis if assisted PD programmes are not available.

Findings from these studies highlight additional factors that should be explored in preference of modality choice when managing older patients. Given that survival may not be the primary focus for many older people, other factors such as depression, maintaining independence, and treatment burden, are even more relevant to explore in this population [71].

## TRANSPLANTATION

Outcomes of transplantation are less favourable with increasing age, particularly when associated with multi-morbidity and/or frailty [8, 9]. Any cognitive impairment can also make it difficult for individuals to engage with the often complex medication schedules and healthcare visit requirements after transplantation, and transplantation itself can exacerbate cognitive decline [72]. People living with frailty, however, can benefit from transplantation with improvements in survival described (compared to dialysis) [73]. In older people, the morbidity associated with transplantation must be considered when assessing outcomes, as this is likely to have a lasting effect on their QoL. The increasing use of extended criteria kidneys for older recipients also needs to be discussed as long-term outcomes including survival and eventual graft function are poorer compared to standard criteria kidneys; live donor kidneys have the best outcomes [74]. In addition, the work-up for transplantation can be arduous so can be an added healthcare burden for individuals whose lives are already dominated by healthcare interactions. Conversations with individuals about transplantation should therefore include realistic expectations of eligibility, outcomes, and how transplantation will or will not affect symptoms and limitations imposed by comorbidities and ageing. There is some evidence that frailty may improve after kidney transplantation, and some individuals may benefit from prehabilitation and/or structured exercise to optimize health and improve outcomes while waiting for and after transplantation [75]. Indeed, aiming for pre-emptive or early transplantation may be the best KRT decision for an older person with minimal comorbidities and low cardiovascular risk, especially if they have the option of a live donor [76].

## ASSISTED DIALYSIS

Availability of assistance for dialysis will greatly influence the choice of KRT modality for an older person. While many older people may choose a home-based treatment there may be a number of barriers, which mean that individuals are unable to undertake home dialysis themselves, including physical and cognitive impairment [77]. Equally, family, caregiver, or healthcare provider perceptions of the risks of home dialysis for an individual may influence decisions around modality choice. These barriers can be overcome by the use of assisted dialysis.

Despite the resurgence of interest in home haemodialysis (HHD) and favourable modelling data as to the potential financial impact of assisted HHD [78], there are very few programmes that are able to deliver either partially or fully assisted HHD [79]. Assisted HHD will not be discussed further as assisted PD is the predominant form of assisted home dialysis available.

Globally, there is a wide variety of models of assisted PD [80]; again, the nature of the model of care, if any, depends on local funding arrangements. Even in Europe, there are wide variations in funding for assisted PD [81], despite increasing evidence for the financial viability and potential cost-savings over in-centre haemodialysis [82]. As a result, assisted PD is frequently not available. Assistance can range from family members, through trained non-healthcare aides to registered nurses working in community or nursing home settings.

Ideally, any model of assisted PD should be flexible to support older people under a range of circumstances. Individuals starting on PD may need assistance either long-term or as a bridge to support independence over a period of weeks to months, particularly where cognitive or language barriers provide surmountable barriers to independence. Equally, assisted PD has a role in supporting individuals who become frail or lose independence temporarily or permanently while on PD. Awareness of the possibility of assistance should circumstances change, while not needed at the start of dialysis, can be an important factor in older individuals choosing PD. Programmes instituting formal assistance models can be shown to improve access to PD for older people and improve uptake of PD [83, 84].

This flexibility should extend to the modality of assisted PD on offer. Historically, the initial models of assisted PD were continuous ambulatory peritoneal dialysis (CAPD) developed in France [85] but the most widespread models on offer are around automated peritoneal dialysis (APD). However, while assisted APD offers the possibility of increased dialysis dose and frequently fewer visits from the assistant, there are challenges around the use of APD in the older person. Specifically, the attachment to a machine overnight which may alarm, combined with restlessness, impaired sleep patterns and cognitive impairment may negatively impact on the QoL on dialysis. As a result, the model of supportive two-exchange CAPD may offer the balance between burden of healthcare visits and reducing the symptom burden associated with ESKD for the frail, older person where APD may be poorly tolerated [86].

Given the wide variation in models of assisted PD, there continues to be a need for comprehensive assessment of an individual's functional status and their ability to undertake the various tasks associated with PD. This needs to occur in conjunction with a healthcare team that can tailor the assisted dialysis prescription to the individual's goals of treatments in line with availability of local models of care. Furthermore, realistic expectation setting around the nature of assisted dialysis and who will undertake which tasks around home dialysis may be needed as part of the education part of any shared decision making.

Ultimately, when choosing KRT modality patients and clinicians need reassurance around potential outcomes. The previously described FEPOD study, has provided robust evidence of comparable outcomes, regardless of modality. The higher treatment satisfaction in patients on assisted PD at enrolment was confirmed in a subsequent longitudinal study [6, 87]. The risk of infection often proves to be a key concern when choosing KRT and can negatively influence perceptions of home dialysis. There is a range of small studies that suggest when compared to unassisted PD there is no change or possibly small increase in risk of PD peritonitis [80], however, reports from large centres with well-established assisted PD programmes suggest lower rates of peritonitis in the setting of assisted PD [88]. Finally, Canadian data suggest hospitalization rates on people on assisted PD is comparable to individuals on in-centre haemodialysis [89].

Access to assisted PD is fundamental as a modality choice for older people choosing KRT; increasing access to these programmes needs to be a key focus with an increasingly older population starting KRT [90].

## SHARED DECISION MAKING

**Figure 1:**
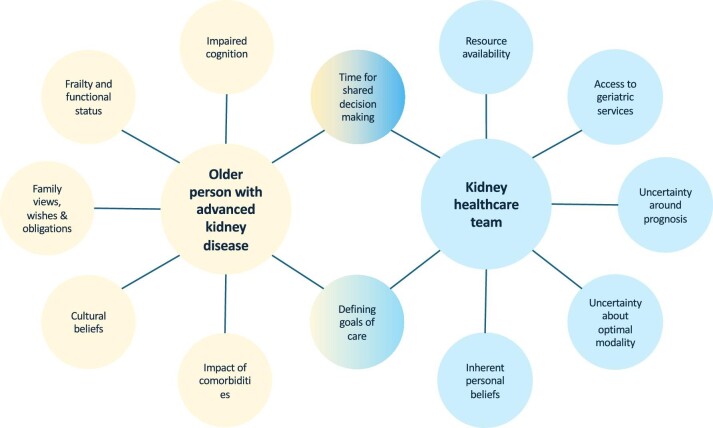
Challenges of decision making for older adults and for the healthcare team.

The challenge in supporting older people in deciding on the optimal KRT approach lies in recognizing that each individual is different with a complex variation of comorbidities (type and clinical stage), level of frailty, social support, lifestyle goals, socioeconomic support, cultural and religious beliefs, and influences. This need of the healthcare provider to know the person they are looking after, underlies the principles of shared decision making (SDM), which is key to achieving person-centred care [91]. As shown in Fig. 1, SDM is a complex sharing of information between the individual about themselves, their concerns and goals and the healthcare team about treatment options, likely trajectories on these treatments and prognosis. An overview of the information exchange that might occur as part of SDM in determining KRT options is detailed in Table 2. SDM is a process and not a one-stop clinic interaction. Getting to know a person during multiple clinical attendances is helpful in determining lifestyle and goals and to start the information process about declining kidney function and the need to make decisions about future management. The benefit of geriatric integration into advanced kidney care has been shown to improve not only immediate but also long-term decision making [37, 92] and there is increasing recognition that this should be more generally available [93]. Geriatric integration also has the benefit of increasing awareness of cognitive impairment, which has been shown to have an impact on decision making capacity [94]. The value of routine geriatric assessment in older people with advanced kidney disease is being assessed in the Netherlands by DIALysis or not: Outcomes in older kidney patients with GerIatriC Assessment (DIALOGICA) study [95, 96].

**Table 2:** Information to share when making decisions about KRT options.

**Older person with advanced kidney disease**	**Healthcare team**
**Daily activities** Spectrum of fully active to mostly housebound Employed or not? Social activities Travel or not? Any dependents e.g. spouse or other family member **Mobility** Walking aids? Speed Falls? **Social support** Lives independently? Needs carers? Who does the shopping, cooking? **Goals** QoL? Length of life? Able to travel overseas? Attend special event? Care for a family member?	**Kidney failure prognosis** Predicted time to dialysis start Symptoms as kidney function declines **Dialysis or not** Realistic description of symptoms, functional status, impact on lifestyle for conservative care **Dialysis at home or in-centre?** Need for transport **Dialysis modality** Unbiased information about processes of HD and PD Impact on symptoms—potential improvement or worsening (e.g. post-HD fatigue) Impact of HD on cognitive function Assistance needed/available for PD? **Overall prognosis** Depends on frailty, comorbidities Dialysis compared to conservative care **Supportive care** Information about conservative care support Symptom control on dialysis

When discussing KRT options, the first decision should be a focus on dialysis or no dialysis, i.e. conservative care. The principles of conservative care were formalized at the Kidney Disease: Improving Global Outcomes (KDIGO) Consensus Conference on supportive care [97]. People who would benefit from conservative care are older people with poor prognosis from comorbidities, poor functional status related to severe frailty and/or cognitive impairment, severe malnutrition, and those whose medical condition precludes technicalities of any type of dialysis [98]. With good supportive care management, QoL is maintained for people opting for conservative care [99]. Patients, however, are often not aware of the option to choose conservative care and may feel pressured by their healthcare professionals to choose dialysis [100].

Despite the general recognition that SDM is the optimal way of determining the best KRT option for older people [101], and the increasing awareness of potential benefits of PD and the impact of HD on accelerated cognitive decline, older people are more likely to be started on HD than PD. In part, this may be due to the non-availability of assisted PD in many countries but is also due to bias against PD in many healthcare systems and by many kidney healthcare teams [102]. Bias is well recognized to occur across all healthcare systems and is related to competing interests, e.g. financial reimbursement favouring HD and not PD in many settings, and poor information or uptake of education [103]. This failure to present evidence-based unbiased information about dialysis modalities and the finding that many nephrologists find it difficult to have the sensitive conversations needed to discuss conservative care [104] will inevitably mean that the best decision regarding KRT options cannot be made. Use of decision aids or staff training manuals may enhance the SDM process, but may not be appropriate for older people particularly with vision or cognitive impairment [105, 106].

Two recent studies have confirmed the importance of unbiased SDM to enable the best decision regarding KRT options for an older person. Using a choice experiment approach, Hole B *et al.* concluded that 327 UK participants (median age 77 years, eGFR 14 ml/min/1.73 m^2^) needed 8%–59% absolute survival benefit 2 years after starting treatment to accept dialysis, with preferences for less frequent treatment and treatment at home [107]. For many, this is not achievable. An observational cohort study using target trial emulation with data from the US Department of Veterans Affairs, 2010 to 2018 has shown that for adults >65 years old starting dialysis when their eGFR fell below 12 ml/min/1.73 m^2^, starting dialysis (predominantly HD) was associated with a gain in overall survival of only 77.6 days compared to those not having dialysis and at the expense of 14.7 fewer days spent at home [108].

A challenge to making the decision about optimal KRT is when to have the conversation. Residual kidney function often declines very slowly in older people [109]. Having discussions about potential kidney failure is stressful to individuals and their families. It is not uncommon for some people to find it very difficult to make a final decision and should be in a bracket ‘deciding not to decide’ [110]. Although many will die before actually needing dialysis, there is also the risk of emergency dialysis if there is a sudden decline in kidney function and no recorded decision for conservative care.

## THE ROLE OF FAMILIES, CULTURE, AND RELIGION

Often overlooked in research studies are concurrent and often hidden psychosocial factors affecting decision making around KRT in older people. These encompass the nature of family and caregiver goals of care, as well as cultural and religious beliefs, particularly when cognitive impairment impacts on capacity for decision making [111]. Given the increasing diversity in those with ESKD and requiring KRT [10], these factors are important for all healthcare teams to acknowledge and achieve ‘cultural competency’, despite the challenges some beliefs can present compared with the focus on patient autonomy as is practised in the English-speaking world and is dominant in the medical literature [112, 113].

Families are frequently heavily involved in SDM around KRT, and often to a greater extent with older people who may be more reliant on their relatives. There may be physical reliance, in that families may need to assist with home-based therapies in those who have dexterity or cognitive issues. Social and emotional support from families is also sought by older people, many of whom may be living with relatives and navigating challenges encountered often in later life, including comorbidity, mobility, social isolation, death of a spouse, and financial instability. It is also important to recognize the impact of caregiver burden particularly when there will be likely functional decline after starting dialysis [62, 114].

The balance in such situations is to involve the family in SDM while maintaining a patient-centred focus in line with professional guidance [115]. Acceptance or rejection of treatments and management plans can vary greatly due to the differing ideas and beliefs across different cultures and religions. For example, while some religions may focus on life-prolonging treatments, others may view such interventions as futile and against the principles of their faith. Some cultures, notably Chinese and East Asian communities, still live by the virtue of ‘filial piety’, which describes beliefs and practices of respect and care for one's parents [113]. The medical implications of this, however, may produce scenarios that clinicians find difficult to navigate; the antidote being early discussion of prognosis and treatment options with involvement of the wider multidisciplinary team.

## CONCLUSION

Deciding the best option for KRT in an individual older person depends on awareness by the individual and their family of all the options, the pros and cons of each and how their own coping with increasing age and comorbidities will affect the outcomes. Figure 1 shows the complex interplay needed between the healthcare team awareness of how the individual is ageing, the ability to discuss prognosis and to give unbiased information about options, including no dialysis.

Finally, as we face an ever older and more co-morbid population, when designing future health services assisted PD needs to be readily available to support older people wanting to receive treatment at home and mitigate the risk of decline in cognitive function associated with haemodialysis.

## Data Availability

No new data were generated or analysed in support of this research.
